# Second-generation proteasome inhibitor carfilzomib enhances doxorubicin-induced cytotoxicity and apoptosis in breast cancer cells

**DOI:** 10.18632/oncotarget.12048

**Published:** 2016-09-15

**Authors:** Yonghua Shi, Yang Yu, Zhenyu Wang, Hao Wang, Shayahati Bieerkehazhi, Yanling Zhao, Lale Suzuk, Hong Zhang

**Affiliations:** ^1^ Department of Pathology, Basic Medicine College, Xinjiang Medical University, Urumqi, Xinjiang 830011, China; ^2^ Department of Pathology, The University of Texas MD Anderson Cancer Center, Houston, Texas 77030, USA; ^3^ Department of Translational and Molecular Pathology, The University of Texas MD Anderson Cancer Center, Houston, Texas 77030, USA; ^4^ Laboratory of Medical Genetics, Harbin Medical University, Harbin, Heilongjiang 150081, China; ^5^ Department of Breast Surgery, The Second Hospital of Jilin University, Changchun, Jilin 130041, China; ^6^ Department of Hepatopancreatobiliary Surgery, the Second Affiliated Hospital of Harbin Medical University, Harbin, Heilongjiang 150086, China; ^7^ College of Public Health, Xinjiang Medical University, Urumqi, Xinjiang 830011, China; ^8^ Texas Children's Cancer Center, Department of Pediatrics, Dan L. Duncan Cancer Center, Baylor College of Medicine, Houston, Texas 77030, USA

**Keywords:** proteasome inhibitor, carfilzomib, doxorubicin, drug resistance, breast cancer

## Abstract

Proteasome inhibition is an attractive approach for anticancer therapy. Doxorubicin (DOX) is widely used for treatment in a number of cancers including breast cancer; however, the development of DOX resistance largely limits its clinical application. One of the possible mechanisms of DOX-resistance is that DOX might induce the activation of NF-κB. In this case, proteasome inhibitors could inhibit the activation of NF-κB by blocking inhibitory factor κB (IκB) degradation. Carfilzomib, a second-generation proteasome inhibitor, overcomes bortezomib resistance and lessens its side-effects. Currently, the effect of carfilzomib on breast cancer cell proliferation remains unclear. In this study, we exploited the role of carfilzomib in seven breast cancer cell lines, MCF7, T-47D, MDA-MB-361, HCC1954, MDA-MB-468, MDA-MB-231, and BT-549, representing all major molecular subtypes of breast cancer. We found that carfilzomib alone had cytotoxic effects on the breast cancer cells and it increased DOX-induced cytotoxic effects and apoptosis in combination by enhancing DOX-induced JNK phosphorylation and inhibiting DOX-induced IκBα degradation. The results suggest that carfilzomib has potent antitumor effects on breast cancer *in vitro* and can sensitize breast cancer cells to DOX treatment. DOX in combination with carfilzomib may be an effective and feasible therapeutic option in the clinical trials for treating breast cancer.

## INTRODUCTION

The proteasome is a protease complex that plays a central role in cellular activities, including the processing of misfolded, unassembled, or damaged intracellular proteins [1]. Its inhibition leads to the accumulation of substrate proteins and, ultimately, cell death [2]. Over the last several decades, proteasome inhibition has been extensively investigated as a selective anti-cancer strategy and validated in clinical trials using first- and second-generation proteasome inhibitors (PIs)[3]. The mechanisms of the antitumor activity of PIs are inhibition of proteasome activity, down-regulation of nuclear factor κB (NF-κB) signaling through blocking inhibitory factor κB (IκB) degradation, induction of cancer cell apoptosis through production of the pro-apoptotic factor tumor necrosis factor alpha (TNF-α), stabilization of p53, and interference with a number of different cell cycle signaling pathways, e.g. AKT, MAPK, and c-Jun [4, 5].

Proteasome inhibition has proven to be an effective therapeutic strategy for multiple myeloma and mantle cell lymphoma [6, 7]. Bortezomib, a first-generation PI, was the first to be approved by the US Food and Drug Administration (FDA) for treatment of multiple myeloma in 2003 and relapsed or refractory mantle cell lymphoma in 2006 [8]. Second-generation PI carfilzomib (CFZ, PR-171) is the second FDA-approved PI for the treatment of recurrent multiple myeloma [9]. CFZ is an irreversible tetrapeptide epoxyketone PI that selectively inhibits the chymotrypsin-like activity of the constitutive 20S proteasome and the immunoproteasome [2]. Compared to bortezomib, CFZ has minimal off-target effects on non-proteasome, serine proteases which is thought to explain its reduced neurotoxicity as compared to that of other PIs [10]. CFZ overcomes bortezomib resistance and lessens side effects in patients treated with bortezomib [9]. These agents have also been studied in solid tumors in a number of phase I and II clinical trials [11, 12], yet they have not shown consistent antitumor activity in solid tumors. At present, the effect of carfilzomib on breast cancer cell proliferation remains unclear.

Breast cancer is a common malignancy affecting women of all ages [13]. While targeted therapy has proven effective in a subset of breast cancer patients, new molecularly targeted agents are necessary for breast cancer patients whose tumors develop resistance to chemotherapy. Doxorubicin (DOX, Adriamycin), an anthracycline, inhibits DNA/RNA synthesis by intercalation between base pairs of DNA strands, inducing apoptosis of tumor cells. Despite the wide use of DOX in cancer treatment, acquired drug resistance and profound cytotoxicity largely limits its clinical application potential [14, 15]. Hence, combined treatment with some sensitizing agent is desirable to overcome the resistance to DOX and increase the antitumor effects. One of the explanations for DOX resistance is that DOX could induce the activation of NF-κB [16]. PIs are known to selectively target cancer cells and make them more sensitive to chemotherapeutic agents. Given the potential for improved efficacy and greater tolerability of CFZ and CFZ's known down-regulation of nuclear factor κB (NF-κB) signaling, we investigated the antitumor activity of CFZ in a panel of molecularly unique breast cancer cell lines alone or in combination with DOX. Here we report that CFZ alone resulted in potent inhibition of cellular proliferation and induction of apoptosis, sensitized cells to DOX-induced toxicity, and intensified DOX-induced apoptosis in combination across a diverse set of breast cancer cell lines *in vitro*.

## RESULTS

### Carfilzomib suppresses the proliferation of breast cancer cells

**Figure 1:**
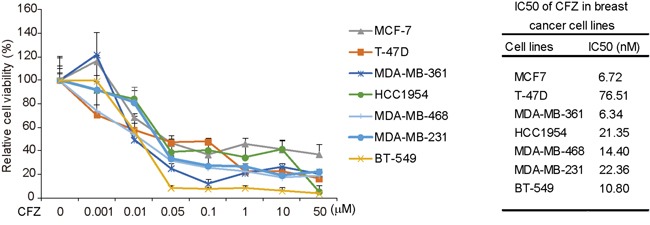
Carfilzomib shows cytotoxic effects on breast cancer cells Cytotoxic effects of carfilzomib on breast cancer cells in MTT assays. Seven human breast cancer cell lines, MCF7, T-47D, MDA-MB-361, HCC1954, MDA-MB-468, MDA-MB-231, and BT-549 were treated with carfilzomib at 0, 0.001 μM, 0.01 μM, 0.05 μM, 0.1 μM, 1 μM, 10 μM, or 50 μM for 72 h, then subjected to MTT assays. The absorbance of each well was measured at 540 nm and plotted for the cell viability curve. IC50 values of carfilzomib in breast cancer cell lines were listed.

To assess the anti-tumor effect of CFZ on breast cancer cells, seven breast cancer cell lines, including MCF7, T-47D, MDA-MB-361, HCC1954, MDA-MB-468, MDA-MB-231, and BT-549 were used, which together represent the major molecular subtypes of breast cancer (Table 1) [17–19]. These cells were treated with CFZ at the indicated concentrations of 0.001 μM to 50 μM for 72 h, then subjected to an MTT assay. The results showed that carfilzomib reduced the cell viability of all types of tested breast cancer cells in a dose-dependent manner (Figure 1). The IC50s of CFZ were between 6.34 nM (MDA-MB-361) and 76.51 nM (T-47D) (Figure 1). The cytotoxic effect of CFZ was confirmed by morphological changes of the cells after treatment for 72 h (Supplementary Figure S1a). Since the IC50s were around the doses of 0.01 μM and 0.05 μM within all cell lines, we only showed the data for these two doses.

**Table 1:** Molecular classification of the human breast cancer cell lines [17–19]

Cell line	Subtype	ER	PR	HER2	Source	Tumor type
MCF7	LuA	+	[+]	−	PE	IDC
T-47D	LuA	+	[+]	−	PE	IDC
MDA-MB-361	LuB	+	[−]	+	P.Br	AC
HCC1954	HER2	−	[−]	+	P.Br	Duc.Ca
MDA-MB-468	BaA	[−]	[−]	−	PE	AC
MDA-MB-231	BaB	−	[−]	−	PE	AC
BT-549	BaB	−	[−]	−	P.Br	IDC, pap

AC, adenocarcinoma; BaA, Basal A; BaB, Basal B; Ca, carcinoma; Duc.Ca, ductal carcinoma; IDC, invasive ductal carcinoma; Lu, luminal; Pap, papillary; P.Br, primary breast cancer; PE, pleural effusion.

ER/PR/HER2 status: ER/PR positivity, and HER2 overexpression (obtained from the Sanger web site) are indicated. Square brackets indicate that levels are inferred from mRNA levels alone where protein data are not available.

To further validate the inhibitory effect of CFZ on cell proliferation, a cellular colony formation assay was performed. The cells were treated with CFZ at the concentrations of 0 μM, 0.005 μM and 0.01 μM for 72 h and then cultured in drug-free medium for about two weeks. Carfilzomib treatment remarkably inhibited the cellular proliferation compared to the control groups (Supplementary Figure S1b). These data indicate that CFZ has a potent inhibitory effect on the proliferation of breast cancer cells, regardless of molecular subtypes.

### Carfilzomib restrains the anchorage-independent growth of breast cancer cells

**Figure 2:**
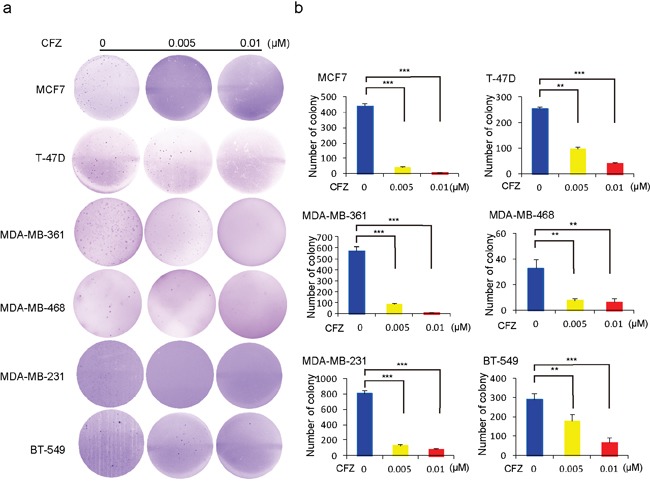
Carfilzomib suppresses the anchorage-independent growth ability of breast cancer cells Cell anchorage-independent growth ability was assessed by soft agar assays. **a.** Six breast cancer cell lines, MCF7, T-47D, MDA-MB-361, MDA-MB-468, MDA-MB-231, and BT-549 were incubated with carfilzomib at 0, 0.005 μM, or 0.01 μM in soft agar plates for three weeks, stained with crystal violet and photographed. **b.** The colonies of (a) were counted and plotted. Data are represented as mean ± SD. * indicates *P*<0.05, ** indicates *P*<0.01, *** indicates *P*<0.001, by *ANOVA* and Dunnett's multiple comparison post-test.

Cancer cells possess the ability to grow into ball-shaped colonies in a three dimensional space when cultured in soft agar. To evaluate whether carfilzomib could impair the anchorage-independent growth ability of breast cancer cells, soft agar assays were performed. Here, breast cancer cells, including MCF7, T-47D, MDA-MB-361, MDA-MB-468, MDA-MB-231, and BT-549, were cultured with CFZ at 0 μM, 0.005 μM and 0.01 μM for three weeks. After that, the visible colonies were fixed and stained. The anchorage-independent growth ability of HCC1954 cell line was too weak to form visible colonies in soft agar, so the data for this cell line is not shown. The number of colonies decreased in CFZ-treated groups compared to the control cells in the tested cell lines (Figure 2a). The inhibitory effects of CFZ on colony formation were dose-dependent and statistically significant in CFZ- treated cells (Figure 2b).

### Carfilzomib induces apoptosis in breast cancer cells

**Figure 3:**
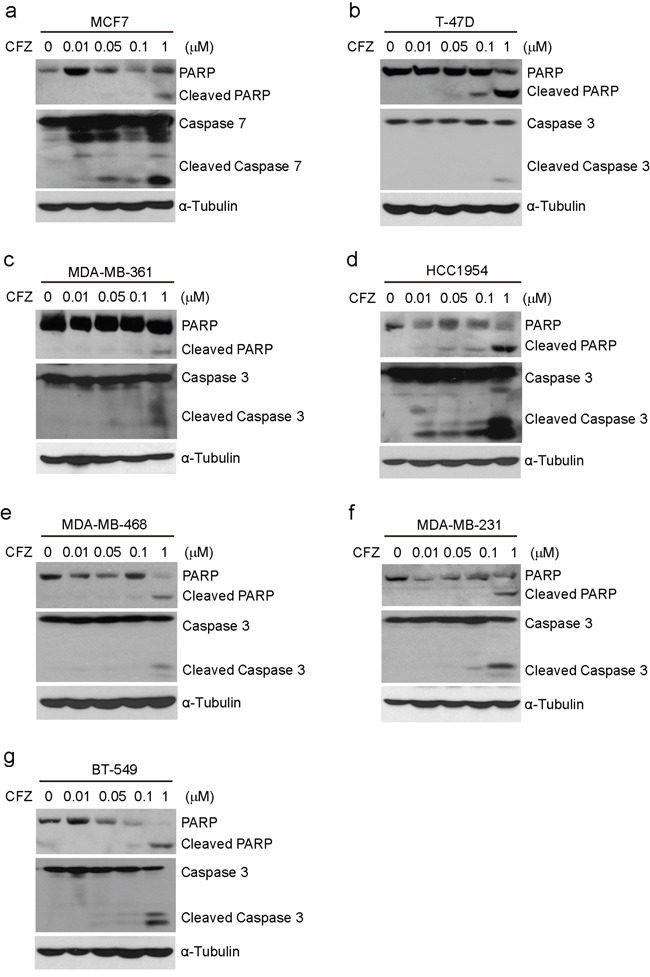
Carfilzomib induces apoptosis in breast cancer cells **a-g.** Breast cancer cell lines MCF7, T-47D, MDA-MB-361, HCC1954, MDA-MB-468, MDA-MB-231, and BT-549 were treated with carfilzomib (0, 0.01 μM, 0.05 μM, 0.1 μM, or 1 μM) for 24 h. Then whole cell lysates were subjected to SDS-PAGE and immunoblotted with antibodies against PARP and Caspase 3 (or Caspase 7) to detect apoptosis. α-Tubulin was used as the loading control.

It has been reported that CFZ can induce apoptosis in a variety of tumor types, including lung cancer, melanoma, and chronic lymphocytic leukemia [11, 20, 21]. To examine whether CFZ could induce apoptosis in human breast cancer cells, the cells were treated with CFZ at concentrations of 0, 0.01 μM, 0.05 μM, 0.1 μM and 1 μM, respectively, and then harvested and subjected to immunoblotting. Since MCF7 cells are Caspase 3 deficient, we tested Caspase 7 as the alternative. We found that CFZ could induce the cleavage of PARP and Caspase 3 (or Caspase 7) in the tested cell lines in a dose-dependent manner except MDA-MB-361, MDA-MB-468 and MDA-MB-231 cell lines (Figure 3a-3g). To further verify that carfilzomib could induce apoptosis in MDA-MB-361, MDA-MB-468 and MDA-MB-231 cell lines, the cells were treated with CFZ at concentrations of 0, 0.05 μM, and 1 μM, respectively, and then harvested and subjected to flow cytometry (Supplementary Figure S2a-S2c). The results showed that CFZ could induce apoptosis in the tested cell lines in a dose-dependent manner. Altogether, the results suggest that carfilzomib alone could trigger apoptosis in breast cancer cells.

### Carfilzomib intensifies the cytotoxic effect of DOX on breast cancer cells

**Figure 4:**
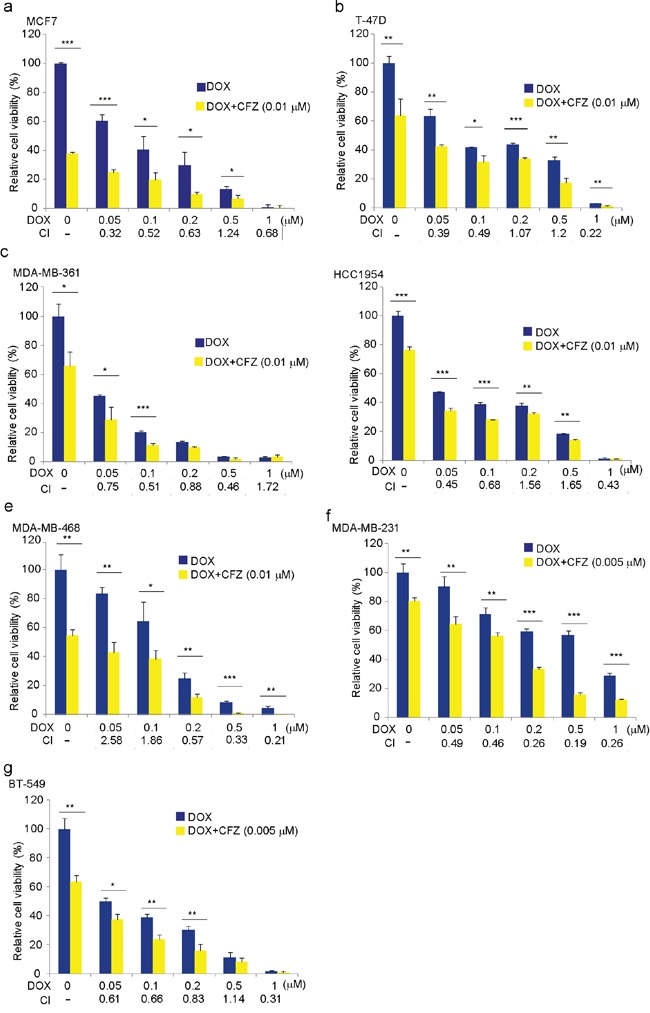
Carfilzomib enhances the cytotoxic effect of DOX on breast cancer cells **a-g.** Breast cancer cell lines MCF7, T-47D, MDA-MB-361, HCC1954, MDA-MB-468, MDA-MB-231, and BT-549 were treated with DOX at the indicated concentrations with or without carfilzomib 0.01 μM or 0.005 μM for 72 h. The cell viability was then measured by MTT assays. CI values were determined using CalcuSyn V2.0 software (BIOSOFT). The data were represented as mean ± SD. *P<0.05, **P<0.01, ***P<0.001, by *t*-test.

To verify whether CFZ and DOX have synergistic effects on breast cancer cells, the cells were cultured in the increased concentration of 0, 0.05 μM, 0.1 μM, 0.2 μM, 0.5 μM or 1 μM of DOX alone or in combination with 0.01 μM of carfilzomib for 72 h, and the cell proliferation was assessed by MTT assay. Cytotoxic effects of 0.01 μM of carfilzomib alone on MDA-MB-231 and BT-549 cell lines were very strong, so we used 0.005 μM of carfilzomib as the alternative. The results showed that the cell viabilities were much lower when treated with the combination compared to those treated with DOX alone (Figure 4a-4g). The combination indexes (CIs) for most combinations were far lower than 1.0, indicating synergistic effects on breast cancer cells (Figure 4a-4g). It implies that CFZ could sensitize the cytotoxicity of DOX on the tested cell lines.

### Carfilzomib enhances the DOX-induced apoptosis in breast cancer cells

**Figure 5:**
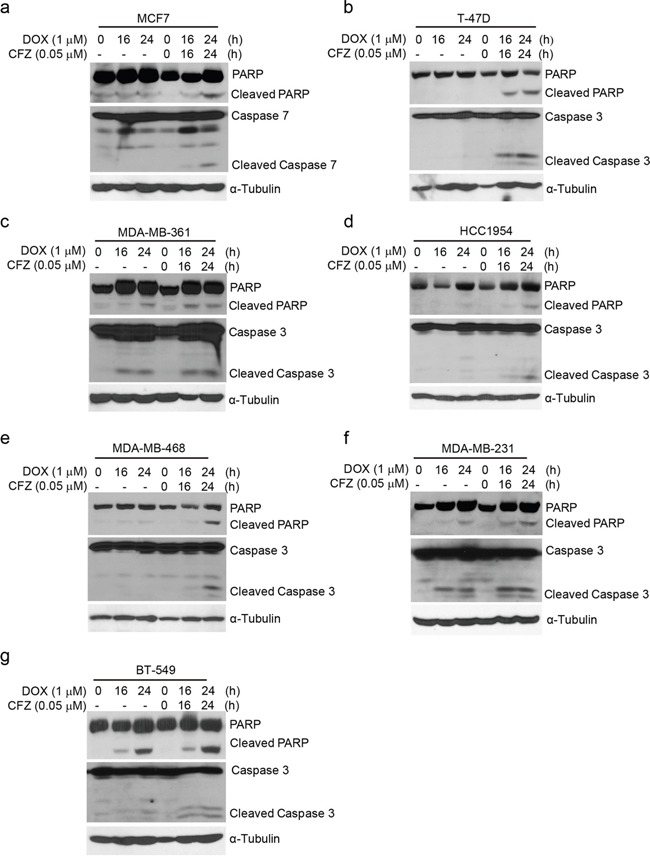
Carfilzomib strengthens DOX-induced apoptosis in breast cancer cells **a-g.** Breast cancer cell lines MCF7, T-47D, MDA-MB-361, HCC1954, MDA-MB-468, MDA-MB-231, and BT-549 were treated with DOX (1 μM) alone or combined with carfilzomib (0.05 μM) for 0, 16 h, or 24 h. Then whole cell lysates were subjected to SDS-PAGE and immunoblotted with antibodies against PARP and Caspase 3 (or Caspase 7) to detect apoptosis. α-Tubulin was used as the loading control.

To explore whether CFZ could strengthen DOX-induced apoptosis in breast cancer cells, the cells were treated with DOX alone (1 μM) or combined with carfilzomib at 0.05 μM (lower dose) for 0, 16 h, or 24 h. Immunoblotting analyses demonstrated that CFZ could boost DOX-induced PARP and Caspase 3 (or Caspase 7) cleavage in all subtypes of breast cancer cell lines tested (Figure 5a-5g). This indicates that CFZ could intensify DOX-induced apoptosis in breast cancer cells.

### Activation of JNK, but not p38 MAPK, and inactivation of NF-κB is required for carfilzomib boosting DOX-induced apoptosis in breast cancer cells

**Figure 6:**
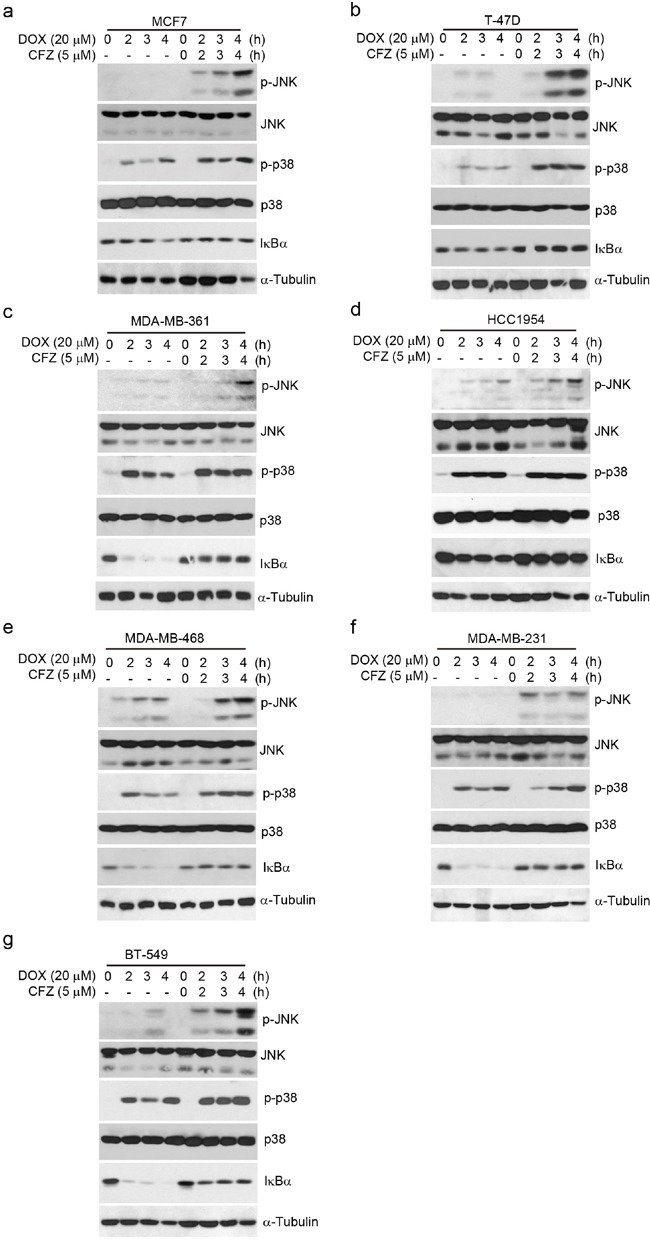
Carfilzomib enhances DOX-induced SAPK/JNK and p38 MAPK phosphorylation and inhibits DOX-induced IκBα degradation in breast cancer cells **a-g.** Breast cancer cell lines MCF7, T-47D, MDA-MB-361, HCC1954, MDA-MB-468, MDA-MB-231, and BT-549 were treated with DOX (20 μM) alone or combined with carfilzomib (5 μM) for 0, 2 h, 3 h or 4 h. Then whole cell lysates were subjected to SDS-PAGE and immunoblotted with antibodies against p-SARP/JNK, SARP/JNK, p-p38 MAPK, p38 MAPK, and IκBα. α-Tubulin was used as the loading control.

Both NF-κB and MAPK are key signal transduction pathways in regulating cell survival [21–24]. Studies have revealed that DOX could induce NF-κB and MAPK activation [16, 25, 26]; meanwhile proteasome inhibitors could inhibit the activation of NF-κB [27]. To better elucidate the potential mechanism of the enhanced effects of carfilzomib on DOX-induced apoptosis in breast cancer cells, we first assessed the effects of the combination of CFZ with DOX on the activity of NF-κB, SAPK/JNK, and p38 MAPK in 7 subtypes of breast cancer cell lines. The cells were cultured with CFZ alone, DOX alone or in combination with CFZ respectively. As shown in Figure 6 and Supplementary Figure S3, CFZ alone could induce SAPK/JNK and p38 MAPK phosphorylation in the tested cell lines. The addition of carfilzomib into DOX up-regulated DOX-induced SAPK/JNK and p38 MAPK phosphorylation, while DOX-induced IκBα degradation was suppressed by carfilzomib (Figure 6a-6g, Supplementary Figure S3a-S3g). These data demonstrate that CFZ reinforced DOX-induced SAPK/JNK and p38 MAPK phosphorylation, whereas it inhibited DOX-induced NF-κB activation.

**Figure 7:**
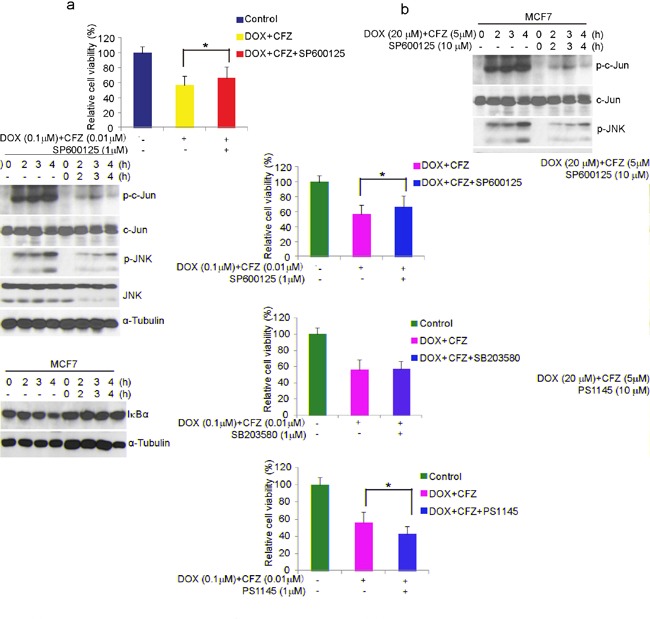
Carfilzomib enhances DOX-induced apoptosis through activation of SAPK/JNK pathway and inactivation of NF-κB in breast cancer cells **a.** MCF7 cells were left untreated or treated with DOX (0.1μM) plus carfilzomib (0.01μM), or in combination with specific pathway inhibitors (SP600125:JNK inhibitor (1μM); SB203580: p38 inhibitor (1μM); PS1145: IKK inhibitor(1μM)) for 48 h, then cell viability was measured by MTT assays. The data were represented as mean ± SD. *P<0.05 was indicated. **b** and **c.** MCF7 cells were incubated with DOX plus carfilzomib, or in combination with SP600125 or PS1145 for the time courses as indicated. Cells were lysed, subjected to SDS-PAGE, and immunoblotted with indicated antibodies. α-Tubulin was used as the loading control.

To investigate which pathway contributes to the enhanced effects of carfilzomib on DOX-induced apoptosis in breast cancer cells, we used specific inhibitors to individually block NF-κB or MAPK pathways. The human estrogen and progesterone receptor-positive MCF7 is a well-established and widely used model system of breast cancer cells. We then examined the impact of the specific inhibitors on the proliferation of MCF7 cells. The results of MTT showed that the JNK-specific inhibitor SP600125, not p38 inhibitor SB203580, protected MCF7 from the enhanced effect of carfilzomib on DOX-induced cytotoxicity, and IκB kinase (IKK) inhibitor, PS-1145 contributed to the enhanced effect of carfilzomib on DOX-induced cytotoxicity (Figure 7a). In order to assess the ability of SP600125 and PS-1145 to block JNK and NF-κB pathways, we performed Western blotting analysis in MCF7 cell line. As expected, carfilzomib in combination with DOX- induced strong phosphorylation of c-Jun and JNK was significantly blocked, and IκBα degradation was blocked when cells were coincubated with SP600125 or PS-1145 (Figure 7b). Furthermore, SP600125 caused significant reduction of carfilzomib in combination with DOX-induced Caspase 7 cleavage, and PS-1145 enhanced to carfilzomib in combination with DOX-induced Caspase 7 cleavage (Figure 7c). Collectively, these studies provide evidence that carfilzomib could boost DOX-induced apoptosis in breast cancer cells by activation of JNK-mediated apoptotic pathway and inhibition of NF-κB.

## DISCUSSION

The clinical success of the first-generation PI Bortezomib as an anticancer therapy for multiple myeloma and relapsed mantle cell lymphoma [28] has bolstered interest in the development of new generations of PIs, such as CFZ and NPI-0052. Development of PIs with distinct substrate selectivity, improved bioavailability and lower toxicity may open the door to widespread usage in solid tumors. Here, we examined the antitumor effect of CFZ on the proliferation of breast cancer cells. We found that CFZ has a high cytotoxic activity, accompanied with reduced cellular proliferation, attenuated colony forming ability, and increased apoptosis in a diverse panel of breast cancer cell lines representing all major molecular subtypes of breast cancers. IC50 values for the tested cell lines treated with CFZ were all in the low nanomolar range from 6.34 nM (MDA-MB-361) to 76.51 nM (T-47D), there was more than 10-fold difference. This is similar to the reported CFZ IC50 values (0.2 nM −99.4 nM) in other solid tumor cell lines [11, 12], and it implies that the different subtypes of breast cancer cell lines might show the sensitivity difference to CFZ. It's worth mentioning that the anchorage-independent growth ability of HCC1954 was too poor to form macroscopic colonies in soft agar. These data suggest that CFZ alone could effectively restrain proteasome activity and has a significant antitumor effect on the tested breast cancer cells. Similarly, studies with CFZ have demonstrated that CFZ causes diminished cell proliferation and increased cell death across a variety of cancer cell lines such as those from lung cancer and anaplastic thyroid cancer [11, 12, 29].

**Figure 8:**
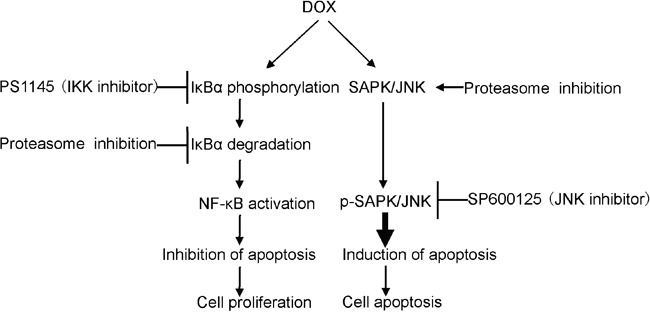
A proposed schematic of carfilzomib's cytotoxic effect on breast cancer The cartoon depicts the proposed signaling events from proteasome inhibition, leading to NF-κB inactivation and SARP/JNK activation in breast cancer cells and, ultimately, cell death.

Increasingly, clinicians are challenged with resistance and toxicity to monotherapy or even in combination therapy. It is reported that DOX induces NF-κB activation and gives rise to DOX resistance in breast cancer and uterine cervical carcinoma [16, 30]. NF-κB activity has been implicated in several aspects of oncogenesis and chemoresistance [31, 32]. These include its ability to induce transcription of genes associated with proliferation and survival, such as cyclin D1, c-IAP-2 and Bcl-xL [33, 34]. Chemoresistance caused by NF-κB activation is a common off-target effect and often necessitates discontinuation of therapy. For these reasons, in recent years, there has been a growing interest aiming at inhibiting NF-κB activity in cancer cells. In addition, DOX also induces the activation of p38 MAPK and JNK apoptosis signaling in breast cancer cells [35]. Combinations of proteasome inhibitors and other chemotherapeutic agents have been examined for use in cancer therapy. In imatinib-sensitive and -resistant chronic myeloid leukemia models, CFZ showed a synergistic effect in combination with tyrosine kinase inhibitors [36]. Dual drug-loaded liposomes containing CFZ and DOX exhibited synergistic efficacy in multiple myeloma *in vitro* and were more efficacious in inhibiting tumor growth *in vivo* [37]. As shown, CIs for most combinations of CFZ and DOX were far lower than 1.0, indicating synergistic effects on breast cancer cells, and the combination of the lower doses of CFZ and DOX significantly and synergistically induced increasingly cytotoxic effects and apoptosis in breast cancer cells by preventing inhibitory factor κB αlpha (IκBα) degradation in the NF-κB signal pathway and activating JNK apoptosis signaling not p38 MAPK in our assays. These data support a model described in Figure 8. It demonstrated that though breast cancer cells showed higher sensitivity to CFZ, breast cancer cells were further sensitized by the combination of CFZ with DOX treatment. CFZ may sensitize breast cancer cells to DOX chemotherapy and thus lessen DOX resistance and toxicity. In kind, some reports suggest that proteasome inhibitors exacerbated DOX-induced cytotoxicity in cardiomyocytes [38] and sensitized leukemia cells and breast cancer cells to DOX by suppressing NF-κB and activating JNK apoptosis signaling pathway [39, 40].

A study that combined treatment of bortezomib and Lapatinib, an inhibitor of epidermal growth factor receptor (EGFR) and HER2 tyrosine kinases, showed a synergistic effect in HER2-overexpressing breast cancer cells [41]. In our work, carfilzomib alone or in combination with DOX showed potent cytotoxic effects and induced apoptosis in HER2-positive MDA-MB-361 and HCC1954 breast cancer cells. CFZ may also be combined with inhibitors to other receptor tyrosine kinases to increase therapeutic efficacy for breast cancer patients. Another paper reported that Nrf2, heme oxygenase 1 (HO-1), and GSH are up-regulated in bortezomib-resistant neuroblastoma [42]. In future studies, the use of PIs and the development of novel strategies to combat cancer that inhibit multiple targets may be indispensable in order to model better treatment options for breast cancer patients.

In conclusion, by using a panel of breast cancer cell lines, we provided compelling evidence that carfilzomib alone could suppress the proliferation and induce apoptosis in breast cancer cells and enhance the cytotoxic effect of DOX and DOX-induced apoptosis in combination by preventing IκBα degradation in the NF-κB signal pathway and activating JNK apoptotic signaling. This study suggests that carfilzomib might serve as an effective drug in the potential combination therapy for breast cancer patients with chemoresistance.

## MATERIALS AND METHODS

### Cell lines and cell culture

The human breast cancer cell lines MCF7, T47D, MDA-MB-361, HCC1954, MDA-MB-468, MDA-MB-231, and BT-549 were obtained from American Type Culture Collection (ATCC, Manassas, VA, USA). MCF7, MDA-MB-361 and MDA-MB-231 cells were routinely cultured in Dulbecco's modified Eagle's medium (DMEM, Lonza, Walkersville, MD, USA), and T-47D, HCC1954, MDA-MB-468 and BT-549 were maintained in RPMI-1640 medium (Lonza), all supplemented with 10% fetal bovine serum (FBS, Sigma-Aldrich Co. LLC. St. Louis, MO, USA), 100 units/ml penicillin, and 100 mg/ml streptomycin. All cells were cultured at 37°C in a humidified atmosphere of 5% CO_2_.

### Antibodies and reagents

The antibodies against IκBα (9242), phospho-SAPK/JNK (Thr183/Tyr185) (9251), SAPK/JNK (9258), phospho-p38 MAP kinase (Thr180/Tyr182) (9211), p38 MAP kinase (8690), PARP (9532), Caspase 3 (9662), Caspase 7 (12827), mouse (7076) and rabbit (7074) were purchased from Cell Signaling Technology (Danvers, MA, USA). The antibody against α-tubulin (10D8) (sc-53646) was purchased from Santa Cruz Biotechnology (Dallas, TX, USA). DOX (D1515) was obtained from Sigma-Aldrich. Carfilzomib (C3022) was purchased from LC Lab (Woburn, MA, USA).

### Cytotoxicity assay

Cell cytotoxicity assays were performed using MTT (MKBH9792V) (Sigma-Aldrich, Spring, TX, USA) following the manufacturer's instructions. Briefly, the cells were seeded in 96-well plates at the density of 5 × 10^3^ cells per well. After 24 h of incubation at 37°C, cells were either allowed to grow in media alone or in media containing increasing concentrations of carfilzomib, DOX, or the combination of the two agents. 72 hours later, cells were observed and photographed by optical microscope. 10 μl of MTT was added into each well, and the cells were incubated for another 2 h. Then, 50μl of DMSO was added into each well, and the cells were incubated for another 10 min. The absorbance of each well was measured at 540 nm and plotted for the cell viability curve. Each experiment was performed in triplicates.

### Determination of synergy

Cells were seeded in triplicate (5 × 10^3^/well) in 96-well plates and allowed to recover overnight. The cells were then treated for 72 h with varying doses of individual drugs alone or varying doses of non-constant ratio of two drugs together. Untreatment was used as a control. Following performance of MTT assays, data were analyzed and combination indexes (CIs) were determined according to the method of Chou and Talalay [43] using CalcuSyn V2.0 software (BIOSOFT). CI values lower than 1.0 were considered evidence of synergism.

### Colony formation assay

Cells were seeded in 12-well plates at 2 × 10^3^ cells per well. 48 or 72 hours later, cells were incubated with carfilzomib at 0, 0.005 μM, or 0.01 μM for 72 h, and then cultured in drug-free medium for about two weeks. After that, cells were fixed and stained with methanol/crystal violet for 10 min and photographed. Each experiment was performed in triplicate.

### Anchorage-independent growth assay

Cell anchorage-independent growth ability was assessed by soft agar assay. In a 6-well plate, the bottom layer was made of 0.5% agar, 2 ml in each well, cooled to semi-solid. For the top layer, cells were mixed with 0.3% agar, 1.5 ml in each well at the density of 5 × 10^3^ cells per well, mixed with carfilzomib at concentrations of 0, 0.005 μM, or 0.01 μM. Cells grew at 37°C for three weeks until the colonies were visible to the naked eye, were stained with crystal violet for 2 h and were photographed. The colonies were counted by Quantity One software (Bio-Rad Laboratories, Inc., Hercules, CA, USA) and plotted. Each experiment was performed in triplicate.

### Immunoblotting

For immunoblotting, after each treatment, cells were washed twice with ice-cold PBS, and spun down. The cell pellets were dissolved in lysis buffer for 30 min at 4°C [50 mM Tris-HCl at pH 7.4, 150 mM NaCl, 1 mM EDTA, 1% NP-40, 0.25% sodium deoxycholate, 1 mM phenylmethylsulfonyl fluoride (PMSF), 1 mM benzamidine, 10 μg/mL leupeptin, 1 mM dithiothreitol (DTT), 10 mM sodium fluoride (NaF), 0.1 mM sodium orthovanadate (OV), phosphatase inhibitor cocktail 2 and 3 (p5726 and p0044, Sigma-Aldrich)]. The solutions were centrifuged at 13,000 rpm for 15 min, and the supernatants were collected as cell lysates. The cell lysates were subjected to 10% or 15% SDS–PAGE electrophoresis and transferred to polyvinylidene fluoride (PVDF) membranes, followed by immunoblotting with the primary antibodies and the horseradish peroxidase-conjugated secondary antibodies against rabbit or mouse IgG. The membranes were developed using the ECL Western blotting system (Thermo Fisher Scientific Inc., Rockford, IL, USA) according to the manufacturer's instructions.

### Flow cytometry and propidium iodide (PI) staining assay

The experiment was performed as the following. Breast cancer cell lines were seeded in 6 cm dishes and treated with carfilzomib of 0, 0.05 μM, or 1 μM for 24 h. Cells were trypsinized, resuspended in the medium with 10% FBS, and centrifuged at 1500rpm for 5 min at 4°C. Cells were then washed with PBS for three times, resuspended in 200 μl of PI staining solution (51-66211E; BD Biosciences), and then transferred into new 5 ml culture tubes with filter. The tubes were gently vortexed and incubated for 15 min at RT (25°C) in the dark, then the samples were analyzed by flow cytometry within 1 h. As viable cells with intact membranes resist PI staining, only the membranes of dead cells are subject to PI staining. Unstained cells were used as a negative control and untreated cells were used as a control for treated cells. Then analysis was performed on a LSR-II flow cytometer (BD Biosciences) using BD FACDiva software v. 6.0.

### Statistical analysis

Statistical analysis was performed using GraphPad Prism 5 software. All values were presented as mean ± standard deviation (SD). *P*-values <0.05 were considered to be statistically significant. Student's *t*-test (two-tailed) or ANOVA (Dunnett's multiple comparison post-test) were used to analyze the difference between the drug treatment groups and control group.

## SUPPLEMENTARY MATERIALS FIGURES



## Abbreviations

PI: proteasome inhibitor
CFZ: carfilzomib
DOX: doxorubicin
DMSO: dimethyl sulfoxide
FDA: Food and Drug Administration
