# Supplementary material for: Second-generation proteasome inhibitor carfilzomib enhances doxorubicin-induced cytotoxicity and apoptosis in breast cancer cells
Source: Oncotarget. 2016 Sep 15;7(45):73697–710. doi: 10.18632/oncotarget.12048 (PMC5342008; doi:10.18632/oncotarget.12048)
Supplement: Supplementary file 1 [file oncotarget-07-73697-s001.pdf]

Second-generation proteasome inhibitor carfilzomib enhances doxorubicin-induced cytotoxicity and apoptosis in breast cancer cells

SUPPLEMENTARY FIGURES

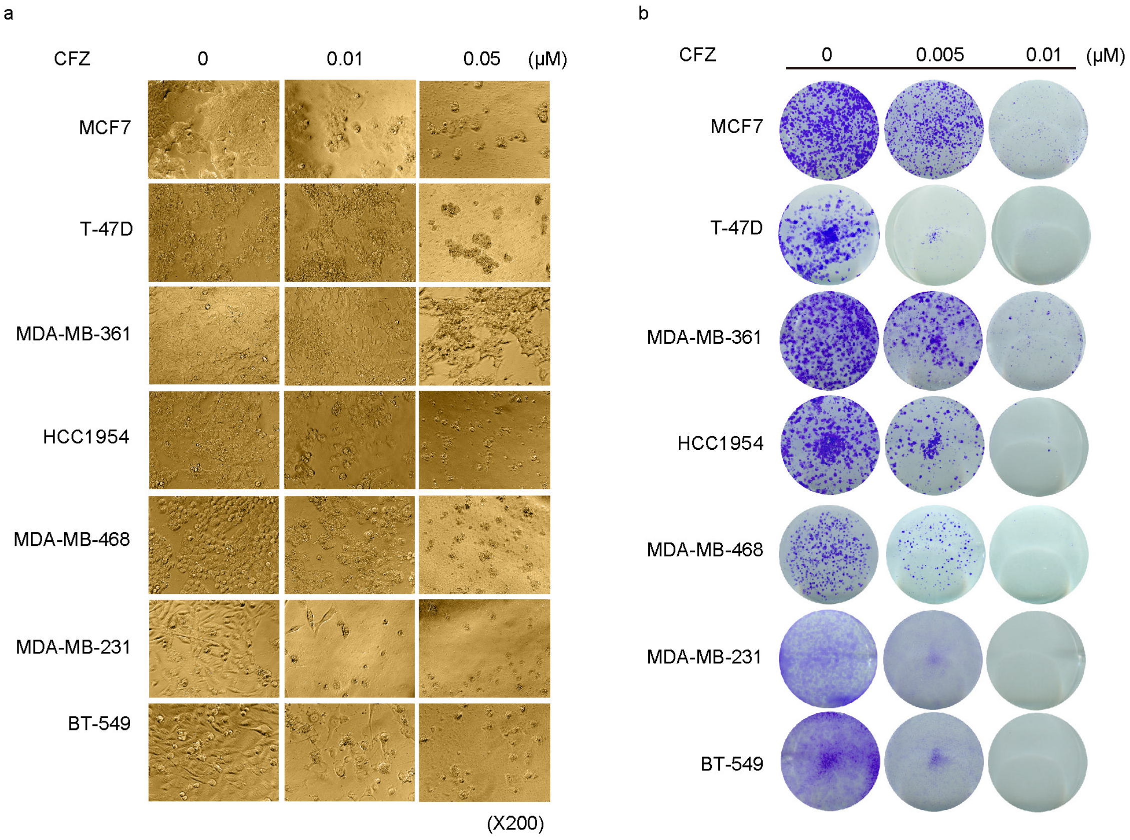

Supplementary Figure S1: Carfilzomib shows cytotoxic effects on breast cancer cells. a. Photographes of cytotoxic effects of carfilzomib on breast cancer cells. Seven human breast cancer cell lines, MCF7, T-47D, MDA-MB-361, HCC1954, MDA-MB-468, MDA-MB-231, and BT-549 were treated with carfilzomib at 0, 0.001 µM, 0.01 µM, 0.05 µM, 0.1 µM, 1 µM, 10 µM, or 50 µM for 72 h, then photographed. Since the IC50s were around the doses of 0.01 µM and 0.05 µM within all cell lines, we only showed the data for these two doses. **b.** Effects of carfilzomib on colony formation of breast cancer cells. Seven breast cancer cell lines were seeded in 12-well plates at 2 $\times 10^3$ per well, then incubated with carfilzomib at 0, 0.005 µM, or 0.01 µM for 72 h and cultured in drug-free medium for about two weeks. The cell colonies were fixed, stained with crystal violet, and photographed.

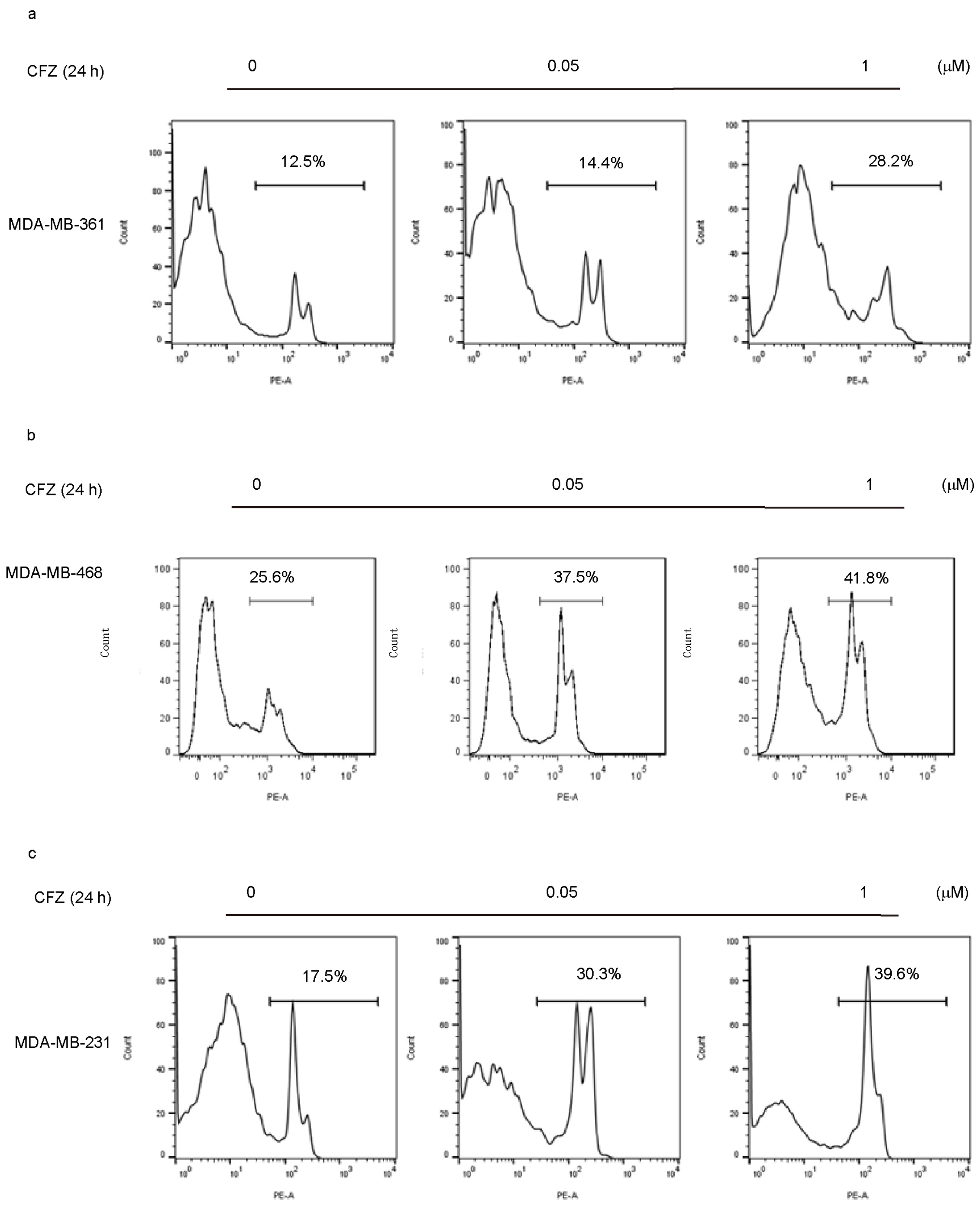

Supplementary Figure S2: Carfilzomib shows cytotoxic effects on breast cancer cells. Flow cytometry of MDA-MB-361, MDA-MB-468 and MDA-MB-231 cells treated with carfilzomib was shown. The cells were incubated in 6-cm dishes at 1×10^6per dish with carfilzomib at 0, 0.05 μM, or 1 μM for 24 h, then analyzed by flow cytometry for the percentage of apoptotic cells by incubating with PI for 15 min at RT.

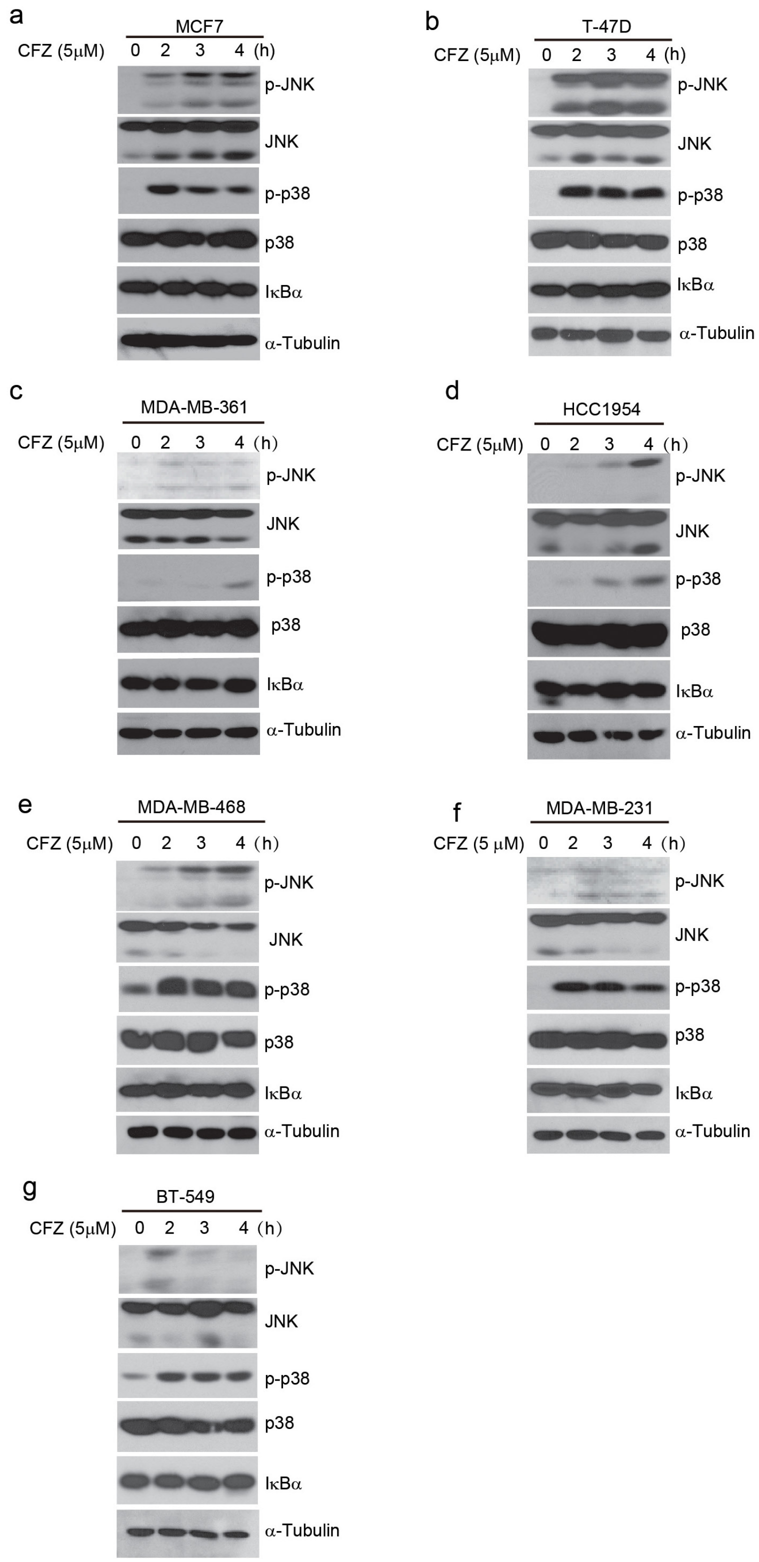

Supplementary Figure S3: Carfilzomib induces SAPK/JNK and p38 MAPK phosphorylation in the tested cell lines. a-g. Breast cancer cell lines MCF7, T-47D, MDA-MB-361, HCC1954, MDA-MB-468, MDA-MB-231, and BT-549 were treated with carfilzomib (5 μM) alone for 0, 2 h, 3 h or 4 h. Then whole cell lysates were subjected to SDS-PAGE and immunoblotted with antibodies against p-SARP/JNK, SARP/JNK, p-p38 MAPK, p38 MAPK, and IκBα. α-Tubulin was used as the loading control.
